# Group-Based Psychological Interventions for Autistic Youth with Chronic Pain: Narrative Review and Practice Considerations for Intensive Interdisciplinary Pediatric Pain Treatment

**DOI:** 10.1007/s10880-026-10138-z

**Published:** 2026-04-11

**Authors:** Jacqueline N. Warner, Leslie A. Sim, Allison Hatley-Cotter, Janette Long, Karen Weiss, Lindsey Vater, Rocco Iacobone, William R. Black

**Affiliations:** 1 Pediatric Psychology, Nationwide Childrens Hospital, Columbus, USA; 2 College of Medicine, Department of Pediatrics, The Ohio State University, Columbus, USA; 3 College of Medicine, Department of Psychiatry and Psychology, Mayo Clinic, Rochester, USA; 4 Levine Childrens Hospital, Charlotte, USA; 5 Center for Autism Spectrum Disorders, Nationwide Childrens Hospital, Columbus, USA; 6 Center for Biobehavioral Health, Nationwide Childrens Hospital, Columbus, USA

**Keywords:** Chronic pain, Autism: group treatment, Intensive interdisciplinary pain treatment, Pediatrics

## Abstract

Emerging work characterizing youth with chronic pain increasingly recognizes a large cohort of youth with co-occurring chronic pain and autism. This development has prompted questions about how to adapt Intensive Interdisciplinary Pediatric Pain Treatment (IIPTs) and the group-based treatments commonly used in these settings to improve accessibility, acceptability, and utility for autistic participants. There is a need for clinically oriented literature that IIPT programs and clinical trialists can use to guide adaptation efforts. Given long-term risks of inadequately treated pediatric pain, we argue it is clinically and ethically important to identify reasonable autism-informed adjustments within existing IIPT frameworks, even as more empirical work unfolds to inform nuance. In this narrative review, we synthesize evidence from pediatric pain psychology and autism intervention literatures to identify overlapping mechanisms and opportunities for adaptation, with a specific focus on group-based CBT/ACT-oriented treatments delivered in IIPTs. We summarize emerging clinical characteristics of autistic adolescents enrolled in IIPTs, bridge autism and pediatric pain group treatment literature, map that literature onto pediatric pain targets and autism-informed IIPT group design considerations and provide practical examples of IIPT group modifications extended from the existing data and the authors’ clinical experience delivering group-based pain psychology services to autistic youth in IIPTs. We also highlight constraints of group formats for autistic youth and emphasize flexible pathways of care.

## Introduction and Overview

Chronic pain affects approximately one in five children and young people with impacts on functioning, mood, and development (Chambers et al., 2024; Eccleston et al., 2003; King et al., 2011). Recent efforts to characterize youth with chronic pain indicate in some programs more than 20% are autistic or display clinically significant autistic features—a rate roughly ten times that observed in the general population (Boerner et al., 2025; Han et al., 2024; Lipsker et al., 2018; Moore et al., 2025; Whitney & Shapiro, 2019; Wiwe Lipsker et al., 2021). This descriptive clarity represents an advancement in pediatric pain, but also raises questions about how to ensure accessibility and appropriateness of treatment. To provide timely recommendations for clinical programs, this paper specifically explores program and treatment considerations for youth with co-occurring chronic pain and autism in the context of group-based care—a common modality of treatment integrated in Intensive Interdisciplinary Pediatric Pain Treatment programs (IIPTS) (ChildKindInternational, 2025).

Autistic youth often have clinical profiles characterized by distinct cognitive, social, and sensory features that are relevant when designing and delivering care for co-occurring chronic pain (Chen et al., 2024; Han et al., 2024; Lage et al., 2024; Mallory & Keehn, 2021; Moore et al., 2025; Sinclair et al., 2020; Wodka et al., 2016). These profiles include facilitators (e.g., pattern recognition, strong memory for routines, interest in structure) and challenges (e.g., sensory modulation differences, slower processing speed, difficulty with rapid turn-taking, intolerance of uncertainty) that shape engagement in group-based care (Han et al., 2024; Sinclair et al., 2020; Stuart et al., 2020). Autistic cognition is broad and heterogeneous (Lord et al., 2020). However, several characteristics are commonly described, including sensory processing atypicality (Pastor-Cerezuela et al., 2020), cognitive inflexibility (Lage et al., 2024; Xie et al., 2020), executive dysfunction (Moore et al., 2025; Wodka et al., 2016), and higher-order cognitive function differences (Alsaedi et al., 2020; Begeer et al., 2014; Jaworski & Eigsti, 2017). In pediatric chronic pain treatment, these features shape how youth perceive and tolerate sensory input (Chen et al., 2024), follow instructions (Mallory & Keehn, 2021), shift between tasks (Lage et al., 2024), and generalize therapy skills to daily life (Prasanna & Nayak, 2024). Early evidence suggests these factors may moderate engagement, functioning, and skill uptake among autistic youth seeking pain services (Han et al., 2024; Sinclair et al., 2020).

These treatment-relevant differences are salient in pain-focused, group-based formats. Manualized group cognitive-behavioral and social skills programs for autistic children and adolescents use predictable routines, structured peer modeling, and in-session rehearsal to support anxiety reduction and social skill gains (Choque Olsson et al., 2017; Reaven et al., 2012a, 2012b). At the same time, autistic youth show high rates of sensory over-responsivity and anxiety linked to sensory and social contexts, which can be intensified in multi-person treatment environments (Bottema-Beutel et al., 2021; South et al., 2017). Early data specifically suggest autistic youth with chronic pain present with a combination of sensory atypicality, cognitive inflexibility, and executive functioning challenges (Han et al., 2024; Moore et al., 2025). This combination may interact with core elements of traditional pain skills delivery models, including in-session skills practice, graded exposure, and between-session homework. Emerging autism-adapted CBT programs for autistic youth (Reaven et al., 2012a, 2012b; Wood et al., 2020) suggest these interactions can either support or hinder skill acquisition, depending on structure and scaffolding. These dynamics underscore the need for autism-informed design rather than assuming group treatment formats, including those used in IIPTs, are equally suitable across neurotypes.

In this paper, we build on the emerging characterization of autistic youth within IIPTs and the group-based dynamics to argue for a focused next step: group-based pain interventions adapted for improved accessibility to young people with co-occurring autism. We draw on emerging data from IIPTs, the existing literature on pediatric pain targets and group treatments, and skill-building structures identified as useful in autism intervention research. Our primary focus is on autistic adolescents with low support needs who can participate in verbally mediated CBT- or ACT-derived pain interventions, recognizing additional and distinct adaptations are likely necessary for autistic youth with higher support needs. We view this focus as a next, but not final, step, since equitable care will require substantially more input from autistic youth and families to optimize individualization, as well as empirical testing to enhance access, utility, and acceptability.

Given the lifetime risks of under- or poorly treated pediatric pain (Campbell, 2022; Friedrichsdorf & Goubert, 2020; Joergensen et al., 2019), timely, evidence-informed adaptation of IIPTs and other pediatric pain treatment settings is warranted, particularly for autistic youth who may experience or communicate pain differently. This paper is guided by the following aims: (1) synthesize who is being served in IIPTs and describe why group treatment can be advantageous in these environments; (2) delineate how specific group-based mechanisms described in the autism literature align with established pediatric chronic pain treatment targets; (3) integrate published evidence and relevant clinical experience delivering group-based pain psychology services within IIPTs to translate these aligned mechanisms into concrete, program-level design choices for existing IIPT group services to further care for autistic youth with co-occurring chronic pain.

We use identity-first language (e.g., “autistic youth”) reflecting published guidelines (Bottema-Beutel et al., 2021) and recommendations of expert consultation. This preference has also been noted in articles involving autistic individuals who can provide self-reported data (Zajic & Gudknecht, 2024), which reflects the individuals primarily considered in context of this paper. Language preference is highly personal; thus, we acknowledge not all autistic people would elect this nomenclature.

## IIPTs: Scope, Outcomes, and Infrastructure Assets

According to the international pediatric pain program registry maintained by ChildKind International, there are upward of 50 interdisciplinary pediatric pain programs in the United States alone; registry data indicate many include an IIPT component (ChildKindInternational, 2025). IIPTs are structured day-hospital or inpatient rehabilitation models integrating medicine, psychology, and PT/OT to restore function, build self-management skills, and normalize activity for youth with high pain-related disability who have not progressed in outpatient care (Claus et al., 2022). Delivered over several weeks (Conroy & Cole-Lewis, 2022; Logan et al., 2012), IIPTs relay improvements in functional disability (Long et al., 2023), school attendance (Randall et al., 2018; Robinson et al., 2019), mood and anxiety (Campanile et al., 2024), and health-related quality of life (Seth et al., 2025), with more variable but often favorable changes in pain intensity (Claus et al., 2022). These effects are reported across single-site cohorts (Campanile et al., 2024), meta-analytic syntheses (Claus et al., 2022), and longitudinal follow-ups (Weiss et al., 2024).

Interdisciplinary pain programs, including IIPTs, have recently become key sites for characterizing autistic youth with chronic pain (Han et al., 2024; Moore et al., 2025). This likely reflects both enhanced capacity for identification and the clinical complexity of youth referred to intensive care. IIPTs incorporate comprehensive, team-based assessment and observation across multiple treatment contexts, which may increase detection and clinical salience of neurodevelopmental differences relative to traditional outpatient models (Conroy & Cole-Lewis, 2022; Hechler et al., 2015). Additionally, youth referred to IIPTs often present with high functional disability and treatment-refractory pain; a profile that may disproportionately include autistic adolescents whose needs have been inadequately addressed (Claus et al., 2022; Moore et al., 2025). Because group-based psychological interventions are a common delivery modality within IIPTs (ChildKindInternational, 2025), these individualized profiles must be supported within shared treatment environments. Accordingly, IIPTs offer a pragmatic platform for autism-informed group adaptation, given their integrated assessment, interdisciplinary coordination, caregiver involvement, and multi-week dosing that allows real-time skills rehearsal and adjustment (Claus et al., 2022; Conroy & Cole-Lewis, 2022; Guite et al., 2018; Russell et al., 2020).

## Group Delivery in IIPTs: Rationale and Mechanisms Relevant to Autistic Youth

Roughly half of U.S. IIPTs report using group-based interventions and similar group-based models are reported in IIPTs in Canada, Australia, New Zealand, Finland, Hungary, Ireland, Spain, the United Kingdom, and the Netherlands (ChildKindInternational, 2025). These groups, along with pain-based group treatment across settings, confer advantages of efficient deployment of relatively scarce clinical resources and are structured to teach active pain coping skills with in vivo, peer-modeled rehearsal to attain functional goals such as school attendance, peer engagement, and family activities (Coakley & Bujoreanu, 2020; Coakley et al., 2018; Darnall et al., 2023; Kashikar-Zuck et al., 2021; Sil & Kashikar-Zuck, 2013). Shared, skills-focused milieus can leverage peer modeling, collective problem-solving, and social-behavioral accountability; unique complements to individual pain-focused encounters (Coakley & Bujoreanu, 2020; Darnall et al., 2023). The group environment is also positioned well to address the social facet of pain conditions for young people. Pain-related stigma, concealment of symptoms, and social isolation (Wakefield et al., 2021; Wolock et al., 2025) are all identified as social challenges for pediatric chronic pain. Group interventions normalizing pain experiences, connecting youth with similar conditions, and incorporating opportunities for healthy peer support to model pain coping and daily living skills are proposed as one method of targeting condition-specific isolation and related psychosocial concerns differently from traditional one-to-one services (Coakley et al., 2018; Nishat et al., 2024; Wakefield et al., 2021, 2022; Wolock et al., 2025).

Similar to pediatric pain research, autism research identifies a clear role for group intervention. When such services are delivered to autistic young people, they typically occur in outpatient mental health or school settings (Gates et al., 2017; Kenworthy et al., 2014; Kilburn et al., 2019). Groups provide structured opportunities to acquire social and coping skills through peer modeling and coached interactions (Chang & Locke, 2016; Gates et al., 2017; Gilmore et al., 2022; McMahon et al., 2013), often augmented by caregiver-assisted formats that support outcomes and home-based rehearsal (Gilmore et al., 2022; Laugeson et al., 2012). Autism literature highlights predictable routines, visual supports, concrete language, and repeated in-session practice (e.g., of socially complex, health- and symptom-related situations) as recommended across group interventions (Laugeson et al., 2012; McMahon et al., 2013), relaying benefits for anxiety reduction (Kilburn et al., 2019) and improved engagement, comprehension, and task persistence (Gates et al., 2017; Gilmore et al., 2022; Kenworthy et al., 2014). These findings briefly highlight empirically grounded mechanisms supportive to autistic youth enrolled in group treatment, and conceptually transferable to pain-focused group intervention.

At the same time, group formats are not uniformly benign. Several autism- and pain-relevant risks warrant explicit consideration. Some adolescents with chronic pain report social invalidation, feeling “different” in peer contexts (Forgeron et al., 2010; Wakefield et al., 2021, 2022; Wolock et al., 2025) and bullying that erodes peer trust (Forgeron et al., 2010; Linkiewich et al., 2023; Wolock et al., 2025). Some autistic adolescents similarly report masking or camouflaging autistic traits in socially demanding group settings, especially when norms prioritize neurotypical communication and behavior (Bottema-Beutel et al., 2021; Spain & Blainey, 2015). Such camouflaging is linked to anxiety, fatigue, and reduced authenticity (Spain & Happé, 2020). When sensory load is high or expectations are poorly scaffolded for autistic participants, group demands may further elicit overwhelm or avoidance rather than engagement (Jordan et al., 2024; Nuske et al., 2025). These constraints suggest group treatment is best conceptualized as one intentional pathway rather than a default, with careful triage, explicit tailoring, and individualized or stepped supports available when group participation is unlikely to be beneficial or acceptable.

Acknowledging both benefits and cautions, several pediatric chronic pain and autism group programs have empirically tested specific social-behavioral mechanisms. Although many pediatric pain groups are delivered in outpatient or non-intensive interdisciplinary settings, we draw on this broader literature to identify group-based mechanisms commonly incorporated within IIPTs. Across programs, group formats demonstrate good feasibility, including high attendance, low attrition, and positive satisfaction, with improvements in functioning, mood, pain catastrophizing, and parent behaviors (Burns et al., 2023; Claus et al., 2022; Coakley et al., 2018; Hechler et al., 2015). In pediatric pain, examples include The Comfort Ability® program, a one-day child and caregiver pain skills workshop with demonstrated feasibility and reductions in pain-related distress (Coakley et al., 2018; Hale et al., 2023), FIT Teens, an outpatient group integrating CBT and exercise that improves disability and mood (Kashikar-Zuck et al., 2012, 2021), and CAPTIVATE, a multi-family outpatient skills group showing early feasibility (Huestis et al., 2017). In autism research, PEERS® and Facing Your Fears demonstrate good feasibility and improvements in social skills, friendship quality, and anxiety (Laugeson et al., 2012; Reaven et al., 2012a, 2012b).

Psychological treatments for pediatric pain are typically CBT- or ACT-based (Kaiser et al., 2015; Sturgeon, 2014). These interventions benefit many adolescents, though responses vary (Fisher et al., 2019), and autistic traits may influence engagement and response (Jordan et al., 2024). Correspondingly, Moore and colleagues (2025) found that youth with more pronounced autistic traits entered IIPTs with greater psychological concerns, social challenges, and family functioning difficulties than peers with less pronounced traits, yet achieved pain-related gains comparable to non-autistic youth. Participants in this study had low support needs and strong verbal and intellectual skills; autistic youth with higher support needs, co-occurring intellectual disability, or significant communication differences may respond differently. Reflexive thematic work with autistic adolescents with chronic pain and low support needs show that core CBT and ACT targets (e.g., functional activation, graded exposure, values-guided goals, cognitive skills, and parent coaching) map well onto commonly endorsed needs, such as predictable structure, concrete and visual supports, sensory-aware environments, and explicit pacing when autism-informed adaptations are used (Jordan et al., 2024). Together, these findings suggest CBT and ACT processes are accessible to a cohort of autistic adolescents in IIPTs—particularly those with lower support needs—while systematic autism-informed adaptations remain essential.

Although IIPTs were not originally designed with autistic accessibility in mind, many structural features, including predictable content and session flow, explicit skills teaching, multimodal learning, and caregiver involvement, align with supports autistic adolescents identify as helpful in CBT and ACT settings (Han et al., 2024; Jordan et al., 2024). Autistic youth and families also describe needs for visual scaffolding, graded activity, sensory-aware environments, and explicit pacing, which parallel existing IIPT practices (Russell et al., 2020; Spain & Happé, 2020; Spain et al., 2023). Together, these data illustrate who is being served in IIPTs and highlight how core group-based processes, including peer modeling, normalization, predictable routines, visual scaffolding, graded activity, and caregiver coaching, map onto established pediatric pain targets and autism-relevant cognitive, social, and sensory profiles (Aims 1–2). Table 1 summarizes this mapping by organizing group mechanisms, their pediatric pain targets, and illustrative autism-relevant adaptations that are feasible within IIPTs and outpatient groups. The next section extends this mapping to outline concrete autism-informed group design considerations for IIPTs.

## Designing Autism-Informed Group Services in IIPTs: Concrete Strategies

Drawing on the mechanisms summarized above, this section highlights elements IIPTs may consider when adapting groups for autistic adolescents whose profiles support participation in CBT- and ACT-oriented protocols (Claus et al., 2022; Hechler et al., 2015). Because empirical trials specifically examining group treatment for autistic youth with chronic pain remain limited, we make recommendations below that synthesize available research with practice-informed observations from the authors’ experiences facilitating pain-focused treatment groups with autistic participants. We present these integrations transparently to distinguish literature-supported findings from hypothesis-generating clinical adaptations. We recommend features that fit IIPT workflows and can be evaluated. Examples reflect practices used within the authors’ pediatric IIPTs and are intended as starting points that can be tailored to local resources and patient populations.

## Staffing and Training

Research on autism interventions emphasizes adequate staffing and autism-specific training as prerequisites for flexible, developmentally attuned care, including group formats (Cooper et al., 2018; Gates et al., 2017). Ideally, groups are co-facilitated by clinicians with complementary expertise in pediatric pain and autism. When this is not feasible, programs can:

1. Identify at least one group leader with dedicated training in autism-affirming practice;
2. Designate staff for one-to-one support if sensory or emotional overload emerges;
3. Enhance autism-informed capacity through didactic education, clinical observation of skilled facilitators, and consultation with relevant experts (e.g., occupational therapists or child development clinicians); and
4. Solicit feedback from IIPT group completers to identify autism-affirming gaps less apparent to program staff.

## Group Membership and Structure/Patient Triage

A recurring clinical question is whether autistic youth with chronic pain are best served through integration into standard pain groups, autism-specific groups, or flexible hybrid models. Available evidence does not support a one-size-fits-all approach. Adolescents with chronic pain frequently report social invalidation, bullying, and feeling “different” in peer contexts, underscoring the importance of intentional group composition to support engagement (Forgeron et al., 2010; Linkiewich et al., 2023; Wakefield et al., 2021, 2022). For autistic youth in IIPTs, triage can balance potential benefits of peer belonging and concrete modeling (Drüsedau et al., 2022; Gates et al., 2017; Zhang et al., 2022) against risks of isolation, bullying, or masking in unsupportive settings (Spain & Blainey, 2015; Wakefield et al., 2021, 2022; Wolock et al., 2025). Our IIPTs use strategies such as:

1. Pre-program materials that outline group processes, social safety norms, and introduce staff;
2. Groups composed to consider developmental, social, and support needs, rather than age or diagnosis alone;
3. Pre-program consultation with existing providers to carry forward familiar communication and coping plans;
4. Opportunities for youth to connect across unstructured and manualized activities to support belonging; and
5. Clear norms that define the group as a psychologically safe, inclusive space that prioritizes mutual respect and individual self-management over camouflaging or suppression of autistic traits or other personal characteristics.

## Group Content & Delivery Adaptations

Evidence from autism and pediatric psychology supports visually scaffolded, predictable skill-building that is concrete, cumulative, graded over time, and incorporates feedback, particularly for executive functioning and coping skills (Kenworthy et al., 2014; Kilburn et al., 2019; Liang et al., 2024). Peer-mediated and group social skill interventions indicate that engaging peers to model adaptive behavior and provide contingent positive reinforcement can increase autistic youths’ social motivation to try new strategies and help normalize pain- and social-related challenges (Chang & Locke, 2016; Kasari et al., 2012; Linkiewich et al., 2023; Wakefield et al., 2022; Zhang et al., 2022). When planning group delivery, emphasis on graduated, collaboratively planned exposure steps that preserve core mechanisms while increasing predictability, graduated pacing, and choice/autonomy over uncertainty, rapidity, and loss of control (Jordan et al., 2024; Kemani et al., 2018; Stuart et al., 2020) may be particularly useful given the growing frequency with which the construct of demand avoidance has been recognized within the framework of autism (Gillberg et al., 2015; Haire et al., 2024; Stuart et al., 2020). Consistent with these findings, we have noticed the utility of the following adaptations within IIPT groups:

1. Pre-enrollment walkthroughs of group spaces;
2. Visual supports (session agendas, step-by-step worksheets, coping menus);
3. IIPT peer graduates as guest speakers, co-facilitators for selected skills, and in-group skill partners;
4. Pain coping plans designed for child-specific real-life situations;
5. Prioritized concrete, child-tailored examples and practice plans over purely conceptual or metaphorical introductions; and
6. Coordinatedg skills practice across group types (e.g., practicing and naming activity pacing during occupational therapy outings)

## Environmental & Sensory Considerations

Sensory sensitivities are a central overlap between autism and chronic pain, with sensory over-responsivity linked to heightened pain and distress (Bar-Shalita et al., 2019; Failla et al., 2018; Jordan et al., 2024). Sensory-immersive accommodations (e.g., immersive virtual reality, sensory-adapted clinical environments) are associated with reductions in pain and anxiety (Cermak et al., 2015; Gold et al., 2021; Stein Duker et al., 2023). With support from occupational therapy, IIPTs can strive for similar outcomes by assessing sensory needs and incorporating accommodations in group spaces while remaining consistent with safety, staffing, and space constraints. Examples include:

1. Offering access to lower-stimulation areas for brief breaks, with clear supervision and messaging that frames breaks as part of self-management practice;
2. Providing in-room sensory modulation options, such as noise-canceling headphones, “step back and observe” spots, sensory-motor tools, and varied seating;
3. Conducting brief sensory regulation rituals at beginning and end of sessions to support transition between therapies; and
4. Integrating neurodivergence considerations and individualized sensory plans into routine interdisciplinary rounds, documentation, and hand-offs.

These practices can be adapted to local resources and tested to balance individual sensory needs with safe, feasible group flow, while also giving autistic youth supported practice in requesting and using accommodations in ways that can plausibly extend to classroom and other real-world group settings.

## Caregiver Integration

Active caregiver participation alongside group is associated with improved outcomes when parents are directly involved in training and implementation (Guite et al., 2018; Nuske et al., 2025; Wolstencroft et al., 2018). For autistic youth with chronic pain, caregiver-focused work within IIPTs may be especially important for supporting generalization of group skills. In our programs, this has included:

1. Teaching parents the pain coping tools taught in child groups;
2. Coaching parents to model coping and reasonable task modification across instances of pain anxiety/avoidance behaviors and sensory discomfort affecting daily functioning;
3. Creating contingency-management scripts tailored to child pain and sensory regulation needs;
4. Collaboratively designing home routines that mirror IIPT patterns (e.g., implementing pacing plans every day, applying communication scripts to enable active pain coping);
5. Offering parent–child skill rehearsal and troubleshooting sessions to promote real-world pain coping generalization; and
6. Coaching caregivers in communicating coping routines and contingency plans to school teams to promote consistency across IIPT and school.

Taken together, the staffing, triage, content, sensory, and caregiver strategies outline autism-informed options that can be layered onto existing IIPT group infrastructures. Table 1 organizes the proposed adaptations by group treatment mechanism and maps each to core pain treatment targets. Illustrative examples of autism-informed modifications are provided to guide clinical implementation and empirical testing.

## Limitations and Future Directions

This narrative review intends to be a pragmatic bridge between the emerging characterization of autistic youth in IIPTs and more established group-based pediatric pain and autism literatures. There are several limitations to acknowledge. First, the review was not conducted as a systematic review or meta-analysis, and we may have missed relevant studies outside the English-language and pediatric pain literatures we prioritized. Second, we did not formally incorporate lived experience consultation in the review process. Although an autism clinical expert on our team reviewed language and recommendations, the ideas synthesized here should be viewed as a beginning framework to be refined and co-designed with youth and families who have lived experience of both autism and chronic pain.

The scope of the current evidence base also constrains the conclusions we can draw. Existing IIPT characterization studies and trials in this domain are limited in number and primarily include autistic adolescents with low support needs who can participate in verbally mediated clinical protocols. Autistic youth with higher support needs, greater communication differences, or co-occurring intellectual disability are under-represented and are likely to require specialized or additional supports that extend beyond what we outline here. Accordingly, our recommendations should not be generalized wholesale to autistic youth with higher support needs, but used to prompt focused work on adaptations and alternative formats for these groups.

Finally, the intervention and design suggestions offered here are largely hypothesis-generating, drawing primarily on observational data or extrapolation from non-pain autism group interventions. Future work should include feasibility and acceptability studies in IIPTs, pragmatic and adaptive designs that accommodate individualized tailoring, and single-case approaches that better capture heterogeneity in response. These efforts should also explore whether traditional pain and functioning measures perform adequately for autistic youth and consider autism-specific outcomes, such as sensory distress or masking-related fatigue. At the same time, evidence linking inadequately treated pediatric pain to persistent disability and long-term mental health risk supports the need for reasonable, autism-informed adjustments now, that are implemented and evaluated iteratively within pediatric pain settings.

See Table 1

**Table 1:** Group mechanisms mapped to pediatric pain treatment targets, with illustrative autism-relevant adaptation examples for IIPT group formats

Mechanism (group delivery feature)	Primary pediatric pain treatment target(s)*	Illustrative adaptation examples for group (autism-relevant; implementable in IIPTs)**
Peer-mediated reinforcement & normalization	Treatment engagement; pain coping skill use; reduced pain-related stigma (Coakley et al., 2018; Darnall et al., 2023)	Deliberate peer matching (grouped for developmental level, social/communication, or other support needs); coached reinforcement in small-group breakouts; non-autistic peer “skill leaders” for group practice; explicit group norms emphasizing respect for diverse communication styles and discouraging teasing or pressure to mask autistic traits (Kasari et al., 2012; Zhang et al., 2022)
Structured repetition and scaffolded learning	Pain coping skills acquisition and generalization (Coakley et al., 2018; Hechler et al., 2015; Kashikar-Zuck et al., 2012)	Consistent and transparent session agenda; cumulative stepwise practice using visual, step-by-step worksheets with clear breakdown of each skill, reinforced by caregivers; brief structured review of previously taught skills and periodic “consolidation” sessions focused on rehearsal rather than new content (Pasqualotto et al., 2021; Coakley et al., 2018; Hale et al., 2023)
Visual & concrete supports	Skills acquisition; task-shifting (Coakley et al., 2018; Nuske et al., 2025)	Visual schedule, coping menu, step-by-step worksheets for pain coping skills; child-specific skills introduction rather than conceptual or metaphorical introduction alone; coping plans specific to child’s various environments rather than general; visual priming for the IIPT group environment via introductory materials and physical walk-throughs before program start (Coakley et al., 2018; Nuske et al., 2025)
Emotional regulation and distress-tolerance supports	Emotional regulation; distress tolerance (Shaffer et al., 2023; Ting & Weiss, 2017; Pielech et al., 2018)	Live modeling and role-play of regulation strategies with coached feedback; explicitly normalizing brief regulation or self-management breaks as part of skill use during or between groups; brief co-regulation rituals at transitions (e.g., beginning- and end-of-group check-ins); parallel caregiver coaching in how to model, cue, reinforce, and communicate about necessary regulation skills to promote pain coping in *specific* child environments (Shaffer et al., 2023; Coakley et al., 2018)
Sensory-aware environment with explicit pacing/break plans	Session tolerability; dose completion; graduated tolerance for activity (Bar-Shalita et al., 2019; Jordan et al., 2024)	Predictable, scheduled micro-breaks; brief menu of supervised in-room self-management options, such as noise-canceling headphones, access to a sensory-motor tool, or moving to a designated low-stimulation spot in the room; transparent “opt-out to observe, then re-enter” plans that specify location, supervision, and re-entry steps; and, when clinically feasible within program safety protocols, use of sensory-adapted spaces or immersive supports for time-limited sensory regulation practice (Cermak et al., 2015; Gold et al., 2021; Jordan et al., 2024)
Graded activity/exposure with person-to-person fit (within group delivery)	Decreased physical activity avoidance and kinesiophobia; decreased functional disability (Kashikar-Zuck et al., 2012; Kashikar-Zuck et al., 2022)	Graded activity or exposure hierarchies tied to functional goals (for example, school attendance, daily routines, and valued activities); explicit labeling and repetition of pacing and gradual activity increases across relevant therapies (e.g., practicing behavioral pain management skills during group PT/OT activities); and adjusting the scale and pace of exposure steps for autistic youth with greater cognitive inflexibility, anxiety, or refusal (Kemani et al., 2018; Sieberg et al., 2017; Christoffersson et al., 2024)
Caregiver involvement via parallel groups (parent psychological flexibility and coaching)	Youth functioning; psychological flexibility; emotional regulation (Sieberg et al., 2017; Pielech et al., 2018; Christoffersson et al., 2024)	Parent psychological flexibility exercises; contingency-management scripts tailored to pain and sensory needs; home routines that mirror child group skills (e.g., pacing plans and communication scripts); joint parent–child skill rehearsals to troubleshoot real-world barriers (Coakley et al., 2018; Lee et al., 2021)
In vivo skills practice and clear home routines	Coping skill use; school participation/return-to-learn (Coakley et al., 2018; Robinson et al., 2019)	In-session rehearsal translated into a visual home plan with micro-goals and tracking; coordination with school-liaison staff to support graded return-to-learn plans that mirror coping skills and routines from group (Guite et al., 2018; Lee et al., 2021)

^*^References in this column primarily derive from pediatric pain group or IIPT studies

^**^References in this column primarily derive from autism and pediatric pain group literature, with practical illustrations of autism-relevant adaptations for IIPT groups

## Data Availability

No datasets were generated or analysed during the current study.
